# Extraction of *Teucrium manghuaense* and Evaluation of the Bioactivity of Its Extract

**DOI:** 10.3390/ijms10104330

**Published:** 2009-11-20

**Authors:** Guihao Yin, Huicai Zeng, Mingjun He, Mingyue Wang

**Affiliations:** 1 Analysis and Test Center of Chinese Academy of Tropical Agricultural Sciences, Haikou 571101, China; 2 Institute of Tropical Bioscience and Biotechnology, Chinese Academy of Tropical Agricultural Sciences, Haikou 571101, China; 3 Hainan Branch Institute of Medicinal Plant Development, Chinese Academy of Medical Sciences & Peking Union Medical College, Wanning 571533, China

**Keywords:** Teucrium manghuaense, extraction, squalene, GC-MS, antioxidant, anti-tumor

## Abstract

The ethanol extract of *Teucrium manghuaense* grown in Hainan province (China) was analysed by GC and GC/MS. Of the constituents 84–96% were identified on the basis of their GC retention times and their mass spectra in regard to authentic compounds. The results revealed that it contained 9-methyl-9-azabicyclo[4.2.1]nonane (7.43%), 2-methylpyrrolidine (19.42%), 3,7,11,15-tetramethyl-2-hexadecen-1-ol (10.84%), and squalene (28.55%), as major components, constituting 66.24% of the extract. The chemical characterization of the ethanol extract by GC/MS also allowed identification of 1-octen-3-one (3.41%), 2-pentyl-piperidine (2.25%), 1-(2-methyl-1-propenyl)-piperidine (4.63%), 2,2′-diethoxy-5,5′-bi-1-pyrroline (0.41%), (*Z,Z,Z*)-9,12,15-octadecatrieniic acid, 2,3-dihydroxypropyl ester (1.56%), vitamin E (2.95%) and stigmasterol (1.39%). Finally, the antioxidant and anti-tumor activities of the ethanol extract have been evaluated. Results show that the extract of *Teucrium manghuaense* leaf possesses strong DPPH^−^, hydroxyl radical scavenging activity and anti-tumor activity.

## 1. Introduction

In the human body superoxide, hydroxyl radicals and hydrogen peroxide are produced by the reduction of oxygen [1]. It is well established that oxygen radicals are involved in various pathological states such as cancer, cardiovascular disorders, arthritis, inflammation, and liver diseases [2,3]. Antioxidants can protect the human body from free radical damage, and prevent cancer and coronary heart disease and aging process [4].

*Teucrium* is a genus of perennial plants, of the family Labiatae. These species are herbs, shrubs or subshrubs, most common in Mediterranean climates. Humans value *Teucrium* as ornamental plants and a source of aromatics, and some species have culinary and/or medicinal value. Several are used as food plants [5]. A common examples for this genus is *Teucrium manghuaense* Sun ex S. Ghow. *Teucrium manghuaense* are rich in essential oils (0.7–4%), and have been proven to show a wide range of biological activities important to human health, for example, antiviral, antitumoral, antiinflammatory and anticoagulant activity [6]. In this study, we extracted bioactive compounds from *Teucrium manghuaense* and characterized the ethanol extract by GC-MS. In addition, we examined the bioactivity of the extract, including antioxidant and anti-tumor activities.

## 2. Materials and Methods

### 2.1. Materials

*Teucrium manghuaense* was obtained from the Spice and Beverage Research Institute at the Xing Long Tropical Botanical Garden of Chinese Academy of Tropical Agricultural Sciences (Hainan province, China). Leaves of *Teucrium manghuaense* was dried (a relative humidity between 2.8% and 3.4%) and ground into fine powder (70 mesh) using a mechanical grinder. Mouse leukemic L1210 cells was obtained from the Shanghai Cellular Institute of China Scientific Academy, Human Colon Carcinoma LoVo Cell Line kindly offered from the Pharmacological Laboratory in Shanghai Jiao Tong University. 1,1-Diphenyl-2-picrylhydrazyl (DPPH) and 3-(4,5-dimethylthiazol-2-yl)-2,5-diphenyltetrazolium bromide (MTT) were purchased from Sigma Chemical Company (St. Louis, MO, USA). Methotrexate (MTX) was purchased from Shanghai Hualian Pharmacy Co Ltd. All other solvents and chemicals were of analytical grade.

### 2.2. Extraction of bioactive compounds

The bioactive compounds of ground *Teucrium manghuaense* leaf were extracted using ethanol as solvent. Extraction was carried out using a shaking incubator at room temperature for 2 h followed by filtration through Whatman No.1 filter paper. The residue was re-extracted twice in the same manner and the three filtrates were combined. The ethanolic extract was concentrated using a rotary evaporator at 55 °C to near dryness. The extraction rate was 4.2%.

### 2.3. GC-MS analyses

The ethanolic extracts compositions were analysed by GC-MS performed using a Trace GC/MS gas chromatograph coupled to an ion trap detector [7]. The fused-silica column was a SLB-5MS silica column (Supelco, Bellafonte, PA, USA) (30 m × 0.25 mm *i.e.,* film thickness 0.25 cm). GC-MS data were obtained using the following conditions: carrier gas helium (He 99.999%); flow rate 1.0 mL·min^−1^; the split ratio 1/70 (v/v). An aliquot of 100 mg of distilled oils were diluted with 1 mL acetone, as were the extracts, and 1.0 μL was injected into the GC-MS system. The oven temperature program was: 70 °C for 13 min, from 70 to 280 °C at 6 °C min^−1^, and holding 280 °C for 16 min. The injector, transfer line and ion trap temperatures were 250, 280 and 200 °C, respectively. The electron impact (70 eV) spectra were recorded at 1 scan/s with a filament emission current of 10 μA. The identification of volatile compounds was based both on comparison of the linear retention indexes (RI) calculated using the Van der Dool and Kratz’s equation with those reported in the literature [8] and by the matching of mass spectra of the compounds with the reference mass spectra of two libraries (Wiley5 and Nist05) coupled with the software of GC-MS and Adams’ library [9]. For the major chromatographic peaks, identification was also confirmed using authentic standards.

### 2.4. Nuclear magnetic resonance

The 400 MHz ^1^H-NMR spectra of all sample batches were analyzed on an AMX-II 600 MHz spectrometer (Bruker Instruments, Inc.), 32 scans were collected into 64 K data points over a spectral width of 4789 Hz (12 ppm) with the transmitter offset at 5.00 ppm, yielding a digital resolution of 0.15 Hz per point. ^1^H-NMR high-temperature spectra at 353 K were measured at 300.13 MHz using a QMP probe. Thirty two scans were collected into 64 K data points over a spectral width of 6172.84 Hz (20.56 ppm). A flip angle of 30° was used. The acquisition time was 5.31 s, followed by a relaxation delay of 1 s. An exponential line broadening window function of 0.3 Hz was used in the data processing. ^13^C-NMR spectra were recorded at 25 °C using a Bruker Avance 600 NH_3_ spectrometer operating at 14 T and equipped with a 5 mm broad-band probe. For the quantitative ^13^C-NMR study the following experimental parameters were used: 90 pulse width (14 s, −3.0 dB), pulse delay of 38 s, spectral width of 21186.44 Hz (120 ppm), 64 K sampled points with 1.5 s to acquire them.

### 2.5. The DPPH radicals scavenging activity

Experiments were carried out according to Kordali *et al.* [10] using a slight modification. Briefly, the extract was diluted with 0.1 mL of solvent. Then 0.5 mM DPPH solution in methanol was prepared, and 0.5 mL of this DPPH solution was mixed with 0.1 mL of various amounts of the extract and vortexed thoroughly. Then, 4 mL of methanol was added to the solution and allowed to stand for 60 min in a dark room. The absorbance was measured at 516 nm using a Shimadzu UV-1601 spectrophotometer (Shimadzu, Kyoto, Japan). Decreasing the absorbance of the DPPH solution indicates an increase in DPPH radical scavenging activity. This activity is given as percent DPPH radical scavenging, which is calculated with the equation:

%DPPH radical scavenging=[(control absorbance− sample absorbance)/control absorbance]×100%.

Control contained 0.5 mL of DPPH solution and 0.1 mL of methanol. Vitamin C at varying concentrations was used as positive control. Data were reported as means ± SD for three replicates.

### 2.6. The hydroxyl radical scavenging activity

The hydroxyl free radical scavenging activity (FRSA) was assayed by using the 1,10-phenanthroline-Fe^2+^ oxidative method [11]. The reaction mixture contained 0.15 mL of 5 mM 2-deoxyribose, 0.4 mL of 0.75 M sodium phosphate buffer solution (PBS, pH 7.4), 0.25 mL of H_2_O, 0.1 mL of 7.5 mM FeSO_4_, 0.1 mL of 1% H_2_O_2_ and 0.1 mL of sample solution. The reaction was started by the addition of H_2_O_2_. After incubation at 37 °C for 1 h, the absorbance was measured at 536 nm. Hydroxyl FRSA was evaluated as the inhibition rate of 1,10-phenanthroline-Fe^2+^ oxidation by hydroxyl radical. The FRSA was calculated using the following equation:

%Hydroxyl radical scavenging=(As−Ac)×100/(Ab−Ac)

where As is the absorbance in the presence of sample and H_2_O_2_; Ac is the absorbance in the presence of H_2_O_2_ without sample; Ab is the absorbance without sample and H_2_O_2_. Each assay was performed in triplicate.

### 2.7. The anti-tumor activity

The MTT assay is a test of metabolic competence based upon assessment of mitochondrial performance, relying on the conversion of the yellow dye 3-(4,5-dimethyl-2-thiazolyl)-2,5-diphenyl-2*H*-tetrazolium bromide (MTT) to the purple formazan derivative by mitochondrial succinate dehydrogenase in viable cells [12]. The cells were incubated with RPMI-1640 medium, supplemented with 10% fetal calf serum, 2 mM glutamine, 100 U/mL streptomycin and 100 U/mL penicillin at 37 °C with 5% CO_2_ before plating in 96-well plates (10^6^ cells/well in 100 μL of medium). After 24 h, the extracts were added, dissolved in DMSO to obtain the final concentrations (100 mg/L, 20 mg/L, 4 mg/L, 0.8 mg/L, 0.16 mg/L). At the end of 72 h incubation, the medium in each well was replaced by fresh medium (200 μL) containing 0.5 mg/mL of MTT. Three hours later, the formazan product of MTT reduction was dissolved in DMSO and absorbance was measured using a micro-plate reader. The effect of extracts was determined as the percentage of reduced dye in the control samples at 590 nm.

## 3. Result and Discussion

### 3.1. GC-MS

**Figure 1.**
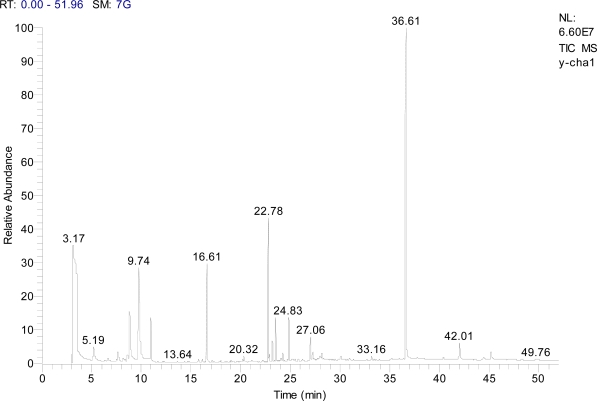
GC–MS chromatogram of ethanolic extract of *Teucrium manghuaense*.

Figure 1 shows the total ion chromatograms (TICs) of the ethanolic extract of *Teucrium manghuaense*. The GC–MS analysis of the ethanolic extract (Table 1) resulted in the identification of 2-methylpyrrolidine, 3,7,11,15-tetramethyl-2-hexadecen-1-ol and squalene, representing 19.42%, 10.84% and 28.55% of the extract, respectively (Table 1). As the ion current generated depends on the characteristics of the compounds concerned it is not a true quantitation. The chemical characterization of the ethanolic extract by GC/MS allowed identification of 1-octen-3-one (3.41%), 2-pentylpiperidine (2.25%), 9-methyl-9-aza-bicyclo[4.2.1]nonane (7.43%), 2-methylpyrrolidine (19.42%), 1-(2-methyl-1-propenyl)-piperidine (4.63%), 2,2′-diethoxy-5,5′-bi-1-pyrroline (0.41%), 3,7,11,15-tetramethyl-2-hexadecen-1-ol (10.84%), (*Z,Z,Z*)-9,12,15-octadecatrienoic acid, 2,3-dihydroxypropyl ester (1.56%), squalene (28.55%), vitamin E (2.95%) and stigmasterol (1.39%) as major components. It also contains other minor constituents, e.g., benzeneethanol (0.82%), 1-(2-pyridinyl)-1-propanone (0.89%), pentadecanoic acid, 14-methyl-, methyl ester (0.62%), (*Z,Z*)-9,12-octadecadienoic acid, methyl ester (0.91%), 5-butyl-2-[1,3-heptadienyl]-pyrroline (0.57%).

### 3.2. NMR

**Figure 2.**
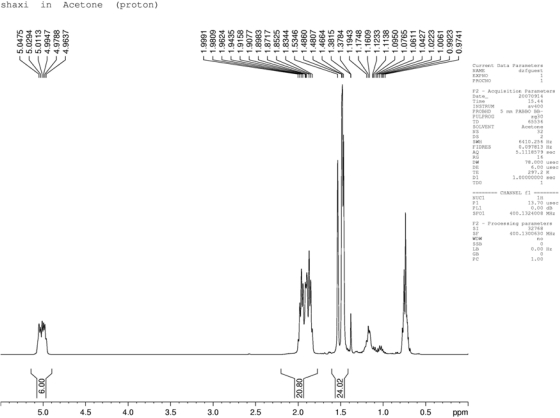
^1^H-NMR spectrum of the ethanolic extract of *Teucrium manghuaense.*

**Figure 3.**
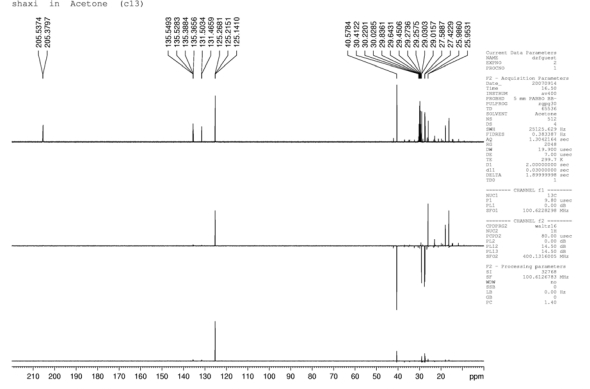
^13^C-NMR spectrum of the ethanolic extract of *Teucrium manghuaense.*

The ^1^H-NMR spectral data (Figure 2) of the extract sample showed that a chemical shift between δ_H_ 4.96 and 5.05 confirm the presence of six vinyl methyls, between δ_H_ 1.83 and 1.99, ten methylenes, and between δ_H_ 1.47 and 1.53 eight methyl groups. The ^13^C-NMR spectral data (Figure 3) of the extract sample showed that a chemical shift between δ_C_ 16.2 and 25.9 confirmed the presence of eight methyls, between δ_C_ 27.4 and 40.6 ten methylenes, and between δ_C_ 125.1 and 135.5 six vinyl methyls. The modified Mosher’s method was carried out based on the chemical shift of the major compound only. Based on the result of the above spectral data, examination of the Δδ values [δ(−) −δ(+)] showed a negative and positive chemical shift distribution to various protons, which can confirm the presence of squalene.

### 3.3. Determination of DPPH radical scavenging capacity

**Figure 4.**
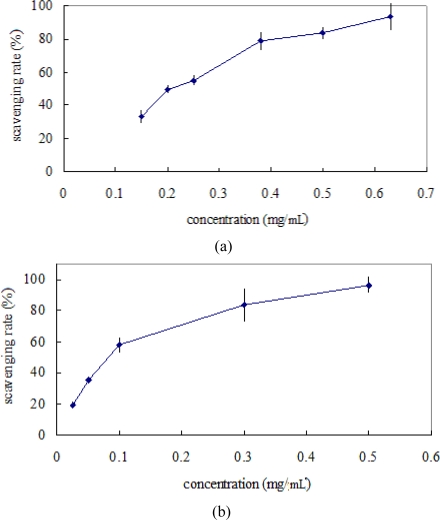
(a) The DPPH radical scavenging activity of ethanolic extract of *Teucrium manghuaense*; (b) The DPPH radical scavenging activity of vitamin C.

DPPH has been widely used to test the ability of compounds or plant extracts to act as free radical scavengers, for example *Teucrium* [13]. Figure 4b shows the percent of DPPH radical scavenging capacity with vitamin C as reference. The experimental data revealed that at various levels the extract was likely scavenge free radicals. From Figure 4a we observed that a dose–response relationship was found in the DPPH radical scavenging capacity; the activity increased as the extract concentration increased. Within concentrations used in the experiment, 0.63 mg/mL ethanolic extract was the strongest scavenger with 93.6%, then 0.5 mg/mL vitamin C as standard with 96.5% (Figure 4b). The 50% inhibition concentration (IC_50_) of the ethanolic extract was determined to be 0.22 mg/mL. From the results, DPPH radical scavenging activity of the extract was weaker than the reference vitamin C at lower concentration. However, it displayed the strong scavenging capacity at higher concentration. Therefore, the ethanolic extract of *Teucrium manghuaense* could act as a natural antioxidant

### 3.4. Determination of hydroxyl radical scavenging activity

**Figure 5.**
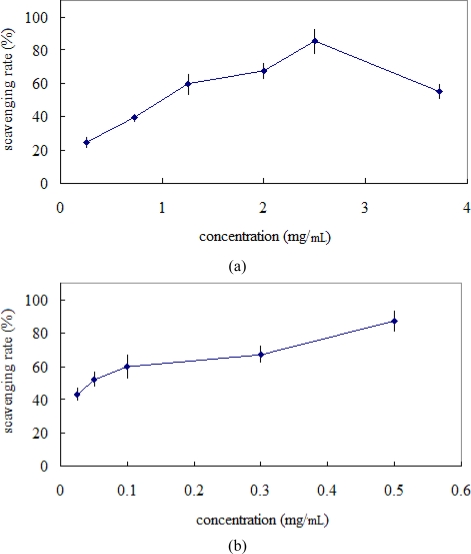
(a) The hydroxyl radical scavenging activity of ethanolic extract of *Teucrium manghuaense*; (b) The hydroxyl radical scavenging activity of vitamin C.

The antioxidant activity of the ethanolic extract of *Teucrium manghuaense* was evaluated by the hydroxyl radical scavenging capacity method. Figure 5b shows the percent of hydroxyl radical scavenging capacity with vitamin C as reference. The activity increased as the concentration increased for the extract. At 0.73 mg/mL, scavenging abilities of ethanol extract of *Teucrium manghuaense* on hydroxyl radicals were 39.3% (Figure 5a). However, the scavenging activity decreased when the concentration of the extract exceeded 2.5 mg/mL. At 0.5 mg/mL, scavenging abilities of vitamin C on hydroxyl radicals were 87.3% (Figure 5b). It indicated that the enthanol extract was less effective in its scavenging ability than vitamin C, which scavenged 87.3% of hydroxyl radicals at 0.5 mg/mL (Figure 5b). However, at 2.5 mg/mL, the extract of *Teucrium manghuaense* showed a scavenging ability of 85.2%.

Cheng, Cui and Chen [14] reported that ethanolic extracts of *Teucrium manghuaense* exhibited good hydroxyl radical scavenging ability and attributed to this the alleged anticarcinogenic properties of *Teucrium manghuaense* extracts. These results indicate that some compounds contained in the ethanolic extracts of *Teucrium manghuaense* are indeed effective scavengers for hydroxyl free radicals. Accordingly, it was anticipated that the high scavenging ability of *Teucrium manghuaense* extracts might confer some antimutagenic properties.

### 3.5. Determination of anti-tumor activity

**Figure 6.**
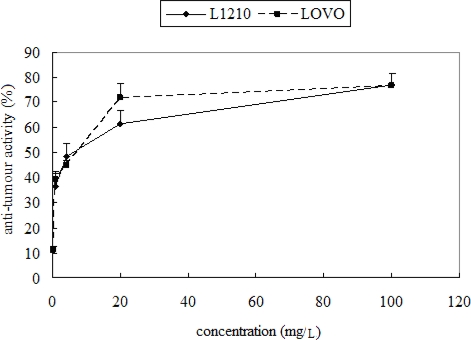
The anti-tumor activity of ethanolic extract of *Teucrium manghuaense.*

Anti-tumor activity of the samples were evaluated using mouse leukemic L1210 cells and human Colon Carcinoma LoVo Cell by enzymatic reduction of MTT (3-(4,5-dimethylthiazol-2-yl)-2,5-diphenyltetrazoliumbromide). As shown in Figure 6, the ethanolic extract inhibited the mouse leukemic L1210 cells and human Colon Carcinoma LoVo Cells growth obviously in a dose-dependent manner. The IC_50_ of the ethanolic extract was 122.8 mg/L on mouse leukemic L1210 cells and 106.0 mg/L on human Colon Carcinoma LoVo Cell, respectively. In contrast to those in the control (methotrexate), the inhibition rate in the ethanolic extract group was significantly lower at all concentrations (p < 0.05). The IC_50_ of methotrexate was 0.41 mg/L on mouse leukemic L1210 cells and 0.26 mg/L on human Colon Carcinoma LoVo Cell, respectively.

## 4. Conclusions

The ethanolic extract of *Teucrium manghuaense* has been analyzed by GC-MS and NMR. GC-MS analysis showed that the components of the *Teucrium manghuaense* extracts are 1-octen-3-one (3.41%), 2-pentylpiperidine (2.25%), 9-methyl-9-azabicyclo[4.2.1]nonane (7.43%), 2-methyl-pyrrolidine (19.42%), 1-(2-methyl-1-propenyl)-piperidine (4.63%), 2,2′-diethoxy-5,5′-bi-1-pyrroline (0.41%), 3,7,11,15-tetramethyl-2-hexadecen-1-ol (10.84%), (*Z,Z,Z*)-(9,12,15-octadecatrienoic acid, 2,3-dihydroxypropyl ester (1.56%), squalene (28.55%), vitamin E (2.95%) and stigmasterol (1.39%), benzeneethanol (0.82%), 1-(2-pyridinyl)-1-propanone (0.89%), pentadecanoic acid, 14-methyl-, methyl ester (0.62%), 9,12-octadecadienoic acid, methyl ester (0.91%), 5-butyl-2-[1,3-heptadienyl]-pyrroline (0.57%). The presence of squalene is further confirmed by the use of NMR. The *Teucrium manghuaense* extracts exhibit a significant antioxidant activity, as determined by removal of DPPH and hydroxyl radicals. In addition, the ethanolic extract of *Teucrium manghuaense* still exhibit a significant anti-tumor activity, as determined by inhibition of L1210 and LOVO cells. These results indicate that there is a potential for developing further studies regarding concentration, chemical characterization, purification, and pharmacological function of the active compounds.

## Figures and Tables

**Table 1.** Compounds identified in the ethanolic extract of *Teucrium manghuaense.*

**No**	**RT**	**compounds**	**CAS #**	**%**
1	5.19	1-octen-3-one	4312-99-6	3.41
2	7.62	2-pentylpiperidine	33354-97-1	2.25
3	8.16	benzeneethanol	60-12-8	0.82
4	8.60	1-(2-pyridinyl)-1-propanone	3238-55-9	0.89
5	8.81	9-methyl-9-azabicyclo[4.2.1]nonane	NA	7.43
6	9.74	2-methylpyrrolidine	765-38-8	19.42
7	10.98	1-(2-methyl-1-propenyl)-piperidine	673-33-6	4.63
8	15.75	2,2′-diethoxy-5,5-bi-1-pyrroline	93042-03-6	0.41
9	22.78	3,7,11,15-tetramethyl-2-hexadecen-1-ol	102608-53-7	10.84
10	24.28	pentadecanoic acid, 14-methyl-, methyl ester	5129-60-2	0.62
11	26.97	(*Z,Z*)-9,12-octadecadienoic acid, methyl ester	2566-97-4	0.91
12	27.06	(*Z,Z,Z*)-9,12,15-octadecatrienoic acid, 2,3-dihydroxypropyl ester	18465-99-1	1.56
13	28.20	5-butyl-2-[1,3-heptadienyl]-pyrroline	139258-00-7	0.57
14	36.62	squalene	7683-64-9	28.55
15	42.01	vitamin E	59-02-9	2.95
16	45.20	stigmasterol	83-48-7	1.39
